# Impact of Glucose Exposure on Outcomes of a Nation-Wide Peritoneal Dialysis Cohort – Results of the BRAZPD II Cohort

**DOI:** 10.3389/fphys.2019.00150

**Published:** 2019-03-05

**Authors:** Vitor Radunz, Roberto Pecoits-Filho, Ana Elizabeth Figueiredo, Pasqual Barretti, Thyago Proença de Moraes

**Affiliations:** ^1^Programa de Pós-Graduação em Ciências da Saúde, Escola de Medicina, Curitiba, Brazil; ^2^Programa de Pós-Graduação em Medicina e Ciências da Saúde (Nefrologia), Pontifícia Universidade Católica do Rio Grande do Sul, Porto Alegre, Brazil; ^3^Faculdade de Medicina de Botucatu, Universidade Estadual Paulista, Botucatu, Brazil

**Keywords:** peritoneal dialysis, cohort, glucose, membrane, death, ultrafiltration failure

## Abstract

**Background:** Data investigating the association of glucose exposure with technique failure and patient survival are limited to retrospective cohorts and was never tested outside Asia and considering the presence of competing risks.

**Methods:** Prospective multicenter cohort study of incident peritoneal dialysis patients where the association of cumulative glucose exposure in 6, 12, and 24 months with patient survival and technique failure was tested using Cox regression analysis and competing risk analysis.

**Results:** We analyzed 4367 incident peritoneal dialysis patients with mean age 59.0 ± 15.8 years, 43.9% were diabetics, 46.7% males and 64.4% Caucasians. Glucose exposure was not associated with patient survival independent of the time of exposure and even after adjustments for confounders. In contrast, higher glucose exposure was associated with more technique failure in the Cox and competing risk models. The higher risk for technique failure was found in the subgroup exposed to the higher amount of glucose to a maximum of 86% in the model analyzing cumulative glucose exposure for 1 year.

**Conclusion:** Glucose exposure was associated with technique failure but not with patient survival.

## Introduction

Glucose has long been used as an osmotic agent in peritoneal dialysis (PD) solutions. Over decades, it was responsible to maintain hundreds of thousands of patients with end-stage renal disease alive and with relatively few symptoms (Li et al., 2017). However, glucose absorption during PD has also been implicated in the development of several metabolic and cardiovascular complications (de Moraes and Pecoits-Filho, 2009; Cho et al., 2010; de Moraes et al., 2015) that eventually lead to the description of higher rates of technique failure and mortality in some studies (Wu et al., 2012; Wen et al., 2015).

The negative impact of glucose exposure on solute transport was first described in the Stoke PD Study (Davies et al., 2001). The chronic exposure of the peritoneal membrane to glucose may cause structural and physiological changes that could potentially increase technique failure and mortality. Nevertheless, only a few relatively small studies evaluated the association of glucose exposure with hard outcomes and all in Asian patients. Wu et al. and Wen et al. in two retrospective studies found that higher glucose exposure was associated with technique failure and cardiovascular (CV) mortality (Wu et al., 2012; Wen et al., 2015). The association of patient mortality with glucose exposure are thought to be in part mediated by a negative impact of glucose on carbohydrate metabolism (Li et al., 2013; de Moraes et al., 2015). However, the incidence of obesity, metabolic syndrome and cardiovascular disease differs considerably across countries (Han and Lean, 2016) and no study of our knowledge investigated the association of glucose exposure on patient survival outside Asia.

Therefore, the aim of this study is to investigate the impact of glucose exposure on patient and technique survival of chronic PD patients of the largest cohort in Latin America.

## Materials and Methods

The BRAZPD II study was a major nation-wide cohort that included patients from December 2004 to January 2011. It collected clinical, demographic and laboratorial data of 9905 adult patients in peritoneal dialysis from 122 centers from all geographic regions across Brazil. This cohort covered around 65% of all PD patients in the country, all of them using standard Baxter^®^ solutions, and icodextrin was not available in Brazil. All data were collected monthly using a specific software (PDNet) designed to the study (de Moraes et al., 2014).

Residual renal function (RRF) was classified at patient inclusion in the study as present (>100 ml/day) or absent. Longitudinal data on RRF and membrane profile were not available. The study protocol was approved by the Ethical Committee of the Pontificia Universidade Católica do Paraná (CEP-PUCPR 448) and the National Ethical Committee under the process 25000.187284/2004-01. All subjects gave written informed consent in accordance with the Declaration of Helsinki.

### Study Population and Outcomes

The inclusion criteria were age >18 years old and start PD within the recruitment period. There were two events of interest: death for any cause and technique failure. The later was defined as a definite transfer to hemodialysis for any reason.

### Glucose Exposure

Glucose exposure was calculated as the product of glucose concentration times the volume of each bag prescribed in a regular dialysis day. For example, in a CAPD patient prescribed with 4 exchanges of glucose 2.5% and 2 l of infusion volume, the glucose exposure would be (4 ml × 2000 ml) × 0.025 = 200 g of glucose/24 h. Then we calculated time-average glucose exposure and divided the population in quartiles.

**FIGURE 1:**
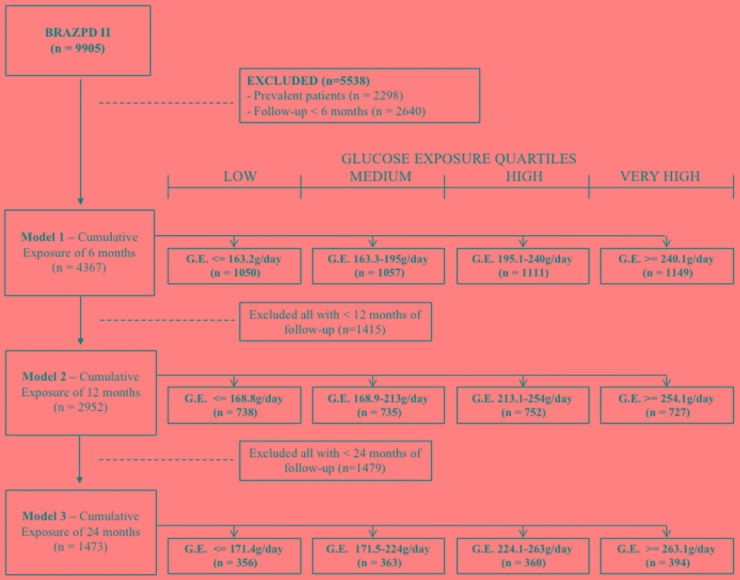
Study design.

In order to evaluate the impact of the cumulative glucose exposure on outcomes we created 3 models. These models represent 3 subgroups of patients based on the time that they remained on PD and the time-average glucose exposure during this period: patients on Model I, II and III remained on PD for a minimum period of time of 6, 12, and 24 months. The first quartile comprises patients with the lowest glucose exposure whilst the 4th are the patients with the highest glucose exposure (Figure 1).

### Statistical Analysis

Continuous variables were expressed as mean ± SD or median and interquartile range, whilst categorical variables were expressed as frequencies and percentages. Patient survival and technique failure were analyzed by the traditional Cox regression and by the competing risk model as proposed by Fine and Gray. Patients alive at the end of the study were always treated as censored. For a better understanding of how we classified censoring and competing risks for both Cox and Fine and Gray models and for the events mortality and technique failure please see Table 1.

**Table 1:** Definition used for censoring, events and competing risks.

	Event	Competing risk	Censored
Cox regression analysis	D	-	TF +T + L + R + A
	TF	-	D + T + L + R + A
Competing risk analysis	D	TF + T + L + R	A
	TF	D + T + L + R	A


*D, death for any cause; TF, technique failure; T, transplantation; L, loss of follow-up; A, alive at the end of the study; R, recovery of kidney function*.

We then tested all potential confounders in an univariate analysis (with and without the presence of competing risks) and those that had a *p*-value < 0.10 were selected for the multivariate model. In addition, we also tested all variables selected for the multivariate model for collinearity (Supplementary Tables S1 and S2).

Assumptions for proportional hazards and proportional sub-distribution hazards were checked with residual plots. Sub-hazard distribution ratios were calculated as proposed by Fine and Gray. Statistical significance was set at the level of *p* < 0.05 and all analysis performed using the software STATA 14^®^.

## Results

**FIGURE 2:**
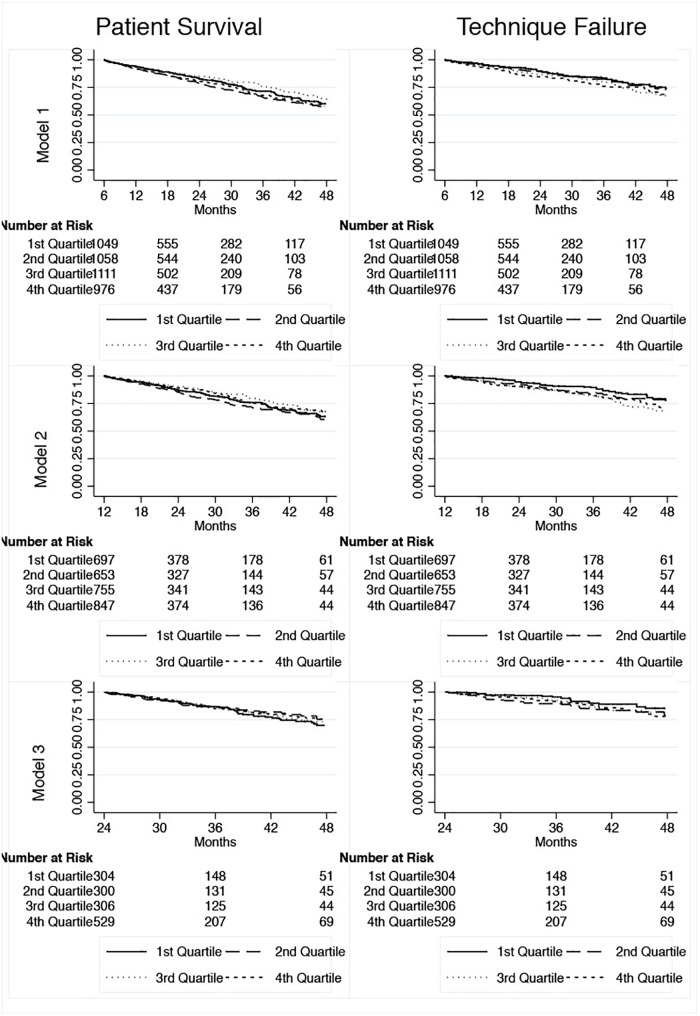
Kaplan-Meier curves for patient survival and technique failure.

We analyzed the outcomes of 4367 incident patients in PD that fulfilled the inclusion criteria. Mean age was 59.0 ± 15.8 years, 43.9% were diabetics, 46.7% males and 64.4% were White. The complete demographic and clinical of our patients are reported on Table 2. Kaplan Meier curves for patient survival and technique failure by quartiles are shown in Figure 2.

**Table 2:** Clinical and demographic characteristics of the study population.

Variable	
Age (years)	59.1 ± 15.8
Body mass index	24.7 ± 4.7
Coronary artery disease (yes)	917 (21.0%)
Diabetes (yes)	1,918 (43.9%)
Gender (Male)	2,041 (46.7%)
Initial Modality (CAPD)^∗^	1,938 (44.4%)
Literacy (<4 years)	1,527 (35.0%)
Peripheral artery disease (yes)	884 (20.2%)
Pre-dialysis care (yes)	2,276 (52.1%)
Previous hemodialysis (yes)	1,780 (40.8%)
Race (White)	2,811 (64.4%)
Residual renal function (yes)	2,937 (67.2%)


*^∗^173 patients with missing information*.

### Patient Survival

There were 755 deaths, of which 278 were cardiovascular events, 246 sepsis not related to the therapy, 78 related to peritonitis, 60 for stroke, 14 for pulmonary edema, 1 for uremia and 78 unknown. The mortality rate was 8.2 (CI 95% 7.6–8.8) deaths per 1,000 patient-months. Regarding technique failure, peritonitis was the main cause of dropout (*n* = 319), followed by ultrafiltration failure (*n* = 85), catheter dysfunction (*n* = 49), refractory exit-site infection (*n* = 11) and 46 for other causes.

For patient survival, the variables identified in the univariate analysis to compose the multivariable model were body mass index, age, literacy, race, diabetes, peripheral artery disease, history of previous hemodialysis and presence of residual renal function at baseline. For technique failure only age and history of previous hemodialysis met the criteria for inclusion in the multivariable model.

Glucose exposure was not associated with patient survival in any of the 3 models even after adjustments for confounders: Model 1 (SHR 0.95; CI 95% 0.86–1.06), Model 2 (SHR 0.87; CI 95% 0.75–1.01) and Model 3 (SHR 0.90; CI 95% 0.73–1.11) (Figure 2); see Table 3.

**Table 3:** Multivariate analysis for patient survival.

	COX		FINE AND GRAY	
Glucose exposure	HR (95% CI)		HR (95% CI)	
	Univariate	Multivariate	Univariate	Multivariate
**Model 1**				
Medium	1.19 (0.99–1.45)	1.21 (1.00–1.47)	1.17 (0.97–1.42)	1.20 (0.96–1.40)
High	0.87 (0.70–1.07)	0.88 (0.67–1.03)	0.83 (0.68–1.03)	0.83 (0.61–0.94)
Very high	1.13 (0.92–1.39)	1.09 (0.84–1.28)	1.05 (0.86–1.29)	1.03 (0.79–1.20)
**Model 2**				
Medium	1.15 (0.90–1.46)	1.18 (0.91–1.46)	1.10 (0.87–1.40)	1.12 (0.86–1.37)
High	0.82 (0.63–1.06)	0.85 (0.55–1.25)	0.76 (0.59–0.98)	0.78 (0.52–0.88)
Very high	0.89 (0.69–1.14)	0.91 (0.66–1.21)	0.82 (0.64–1.05)	0.84 (0.63–1.05)
**Model 3**				
Medium	0.85 (0.57–1.27)	0.89 (0.62–1.29)	0.80 (0.54–1.19)	0.89 (0.62–1.29)
High	0.91 (0.61–1.34)	1.04 (0.65–1.35)	0.87 (0.59–1.27)	0.91 (0.64–1.31)
Very high	0.88 (0.62–1.24)	0.91 (0.49–1.14)	0.83 (0.59–1.16)	0.86 (0.49–1.04)


*Adjusted for age, previous hemodialysis, race, literacy, peripheral artery disease, diabetes and baseline residual renal function*.

All models for patient survival were adjusted for body mass index, age, literacy, race, diabetes, peripheral artery disease, history of previous hemodialysis and presence of residual renal function at baseline (all these covariates included in the final model for being significantly associated with mortality in the univariate analysis).

### Technique Failure

**FIGURE 3:**
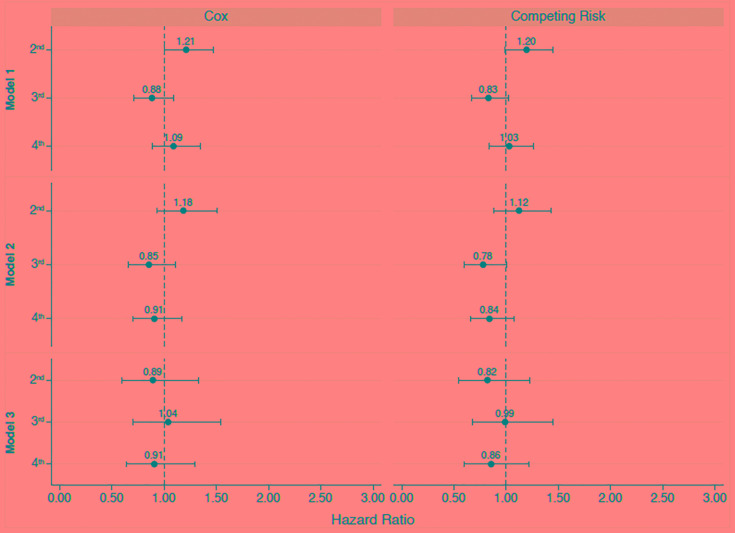
Multivariate analysis for patient survival according to the quartiles of glucose exposure. 1st, first quartile; 2nd, second quartile; 3rd, third quartile; 4th, fourth quartile. Reference values available in Figure 1. *Model 1*: Time-average glucose exposure for the first 6 months of therapy; *Model 2*: time-average glucose exposure for the first l year; *Model 3*: time-average glucose exposure for the first 2 years of peritoneal dialysis.

**FIGURE 4:**
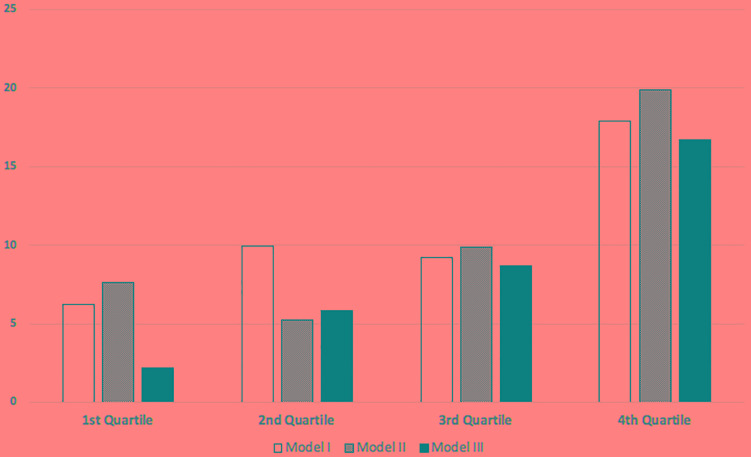
Percentage of ultrafiltration failure as the cause of technique failure stratified by quartiles of glucose exposure.

In contrast to patient survival, glucose exposure was associated with technique failure (Figure 3). Models were adjusted for age and history of previous hemodialysis, the only two variables associated with technique failure in the univariate analysis. In addition, the incidence of ultrafiltration failure as the cause of the technique failure doubled comparing patients from the first to the last quartile of glucose exposure (Figure 4).

#### Model I – Glucose Exposure During the First 6 Months of Therapy

The technique failure rate was 5.6 (CI 95% 5.1–6.0) events per 1,000 patient-months. In the Cox regression analysis, patients from the second, third and fourth quartile had, respectively, a hazard ratio of 1.11 (CI 95% 0.86–1.43), 1.38 (CI 95% 1.08–1.77), and 1.46 (CI 95% 1.13–1.88) compared to patients from the first quartile.

#### Model II – Glucose Exposure During the First Year of Therapy

For 2,952 patients that survived for more than a year, 212 were transferred to HD due to unsuccessful treatment of a peritonitis episode, 56 for ultrafiltration failure, 31 for catheter dysfunction, 10 for refractory exit-site infection and 33 for other causes.

Comparing the four groups we found, in the Cox regression, that patients from the second, third and fourth quartile had a higher risk for technique failure compared to patients from the first quartile: 2nd (HR 1.41; CI 95% 1.01–1.96), 3rd (HR 1.86; CI 95% 1.36–2.55), and 4th (HR 1.63; CI 95% 1.19–2.23).

#### Model III – Glucose Exposure During the First 2 Yearsof Therapy

There were 1,439 patients with more than 2 years of PD and 158 were transferred to HD. Peritonitis remained as the main cause of TF (*n* = 97), followed by ultrafiltration failure (*n* = 23), catheter failure (*n* = 11), refractory exit-site infection (*n* = 7) and other causes (*n* = 20). The number of events was lower in this model but the 2nd (HR 1.74; CI 95% 1.05–2.90) and 4th (HR 1.69; CI 95% 1.06–2.69) quartile remained associated with TF. The 3rd quartile was not statistical significant (HR 1.60; CI 95% 0.95–2.69).

**FIGURE 5:**
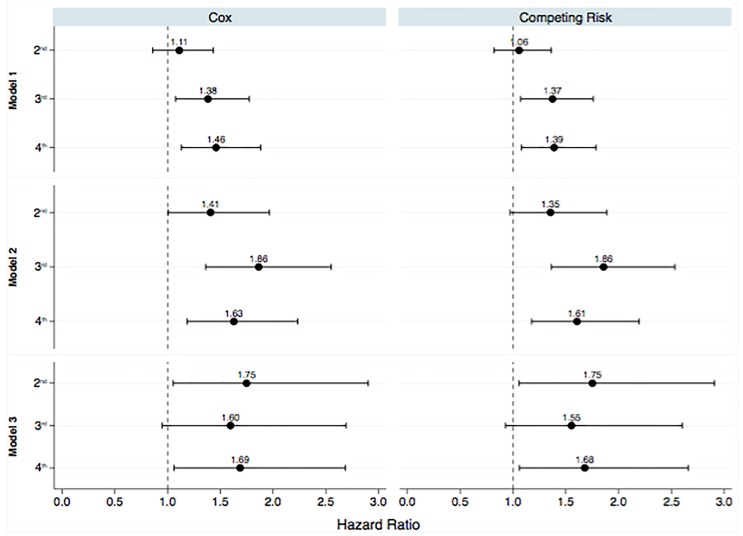
Multivariate analysis for technique failure according to the quartiles of glucose exposure. 1st, first quartile; 2nd, second quartile; 3rd, third quartile; 4th, fourth quartile. Reference values available in Figure 1. *Model 1*: Time-average glucose exposure for the first 6 months of therapy; *Model 2*: time-average glucose exposure for the first l year; *Model 3*: time-average glucose exposure for the first 2 years of peritoneal dialysis.

When we considered the presence of competing risks all associations found in the Cox Regression remained significant (Table 4 and Figure 5).

**Table 4:** Multivariate analysis for technique failure.

	COX		Competing risk	
Glucose exposure	HR (95% CI)		HR (95% CI)	
	Univariate	Multivariate	Univariate	Multivariate
**Model 1**				
Medium	1.13 (0.87–1.46)	1.11 (0.84–1.40)	1.08 (0.84–1.39)	1.06 (0.80–1.33)
High	1.36 (1.06–1.75)^∗^	1.38 (1.09–1.79)^∗^	1.35 (1.06–1.73)^∗^	1.37 (1.08–1.77)
Very high	1.48 (1.15–1.91)^∗^	1.46 (1.13–1.88)^∗^	1.41 (1.09–1.81)^∗^	1.39 (1.08–1.78)
**Model 2**				
Medium	1.42 (1.01–1.98)^∗^	1.41 (1.05–1.94)	1.37 (0.98–1.91)	1.35 (0.98–1.87)^∗^
High	1.84 (1.34–2.52)^∗^	1.86 (1.44–2.58)^∗^	1.84 (1.35–2.51)^∗^	1.86 (1.27–2.52)^∗^
Very high	1.64 (1.19–2.24)^∗^	1.63 (1.24–2.28)^∗^	1.62 (1.19–2.21)^∗^	1.61 (1.08–2.22)^∗^
**Model 3**				
Medium	1.76 (1.06–2.92)^∗^	1.75 (1.05–2.90)^∗^	1.75 (1.05–2.91)^∗^	1.75 (1.04–2.95)^∗^
High	1.53 (0.91–2.58)	1.60 (0.95–2.69)	1.50 (0.90–2.52)	1.55 (0.90–2.60)
Very high	1.70 (1.07–2.71)^∗^	1.69 (1.06–2.69)^∗^	1.69 (1.06–2.67)^∗^	1.68 (1.04–2.68)^∗^


*Adjusted for age and history of previous hemodialysis; ^∗^*p* < 0.05*.

## Discussion

Peritoneal glucose exposure was first linked to abnormal changes in the peritoneal membrane transport in a cohort of 22 patients treated continuously for 5 years in early 2000s (Davies et al., 2001). These abnormalities cause an increase in the membrane permeability which could lead to a faster absorption of glucose reducing the osmotic power of the PD solution and consequently contributing to technique failure and increased risk of death for fluid overload (Grodstein et al., 1981; Churchill et al., 1998; Szeto et al., 2007). However, only few studies tested in large scale the impact of different levels of peritoneal glucose exposure on technique failure and patient survival (Wen et al., 2015). This is the largest cohort study to test the association of glucose exposure with patient survival and technique failure.

Peritoneal dialysis patients may absorb huge amounts of glucose depending of factors as the patient’s membrane transport, dwell time and, mainly, the concentration of glucose in the dialysis solution. In early eighties, Grodstein et al. demonstrated an absorption of up to 350 g per day when CAPD patients were exposed to a high glucose load with 4.25% glucose PD solution (Grodstein et al., 1981). One of the consequences of the chronic exposure to glucose solutions are disturbances in the metabolism of carbohydrate as insulin resistance, dyslipidemia, higher needs of insulin in diabetic patients and even an elevation in fasting glucose and HbA1C in non-diabetic patients (de Moraes and Pecoits-Filho, 2009; de Moraes et al., 2011; de Moraes et al., 2015).

Higher amounts of glucose are often need for high transporters patients in order to increase ultrafiltration and the increased mortality of this subgroup of patients was in the past attributed at least in part to difficulties in the control of fluid overaload (Churchill et al., 1998). However, when fast transporters are properly managed with short cycles in automated PD such high mortality rates tend to disappear (Yang et al., 2008). The majority of our patients were treated on APD and this may have contributed to our finding of a lack of association between glucose exposure and patient survival. Nevertheless, we didn’t capture data on volume status which would be valuable to understand our findings but at the same time very difficult to perform in large cohorts. In addition, we don’t have data on cholesterol and HbA1c levels to evaluate their association with glucose exposure. At the same time, it is interesting to note that the majority of studies that investigated the impact of glycaemia or dyslipidemia control in PD patients were unable to found any association in terms of patient survival (Chawla et al., 2010; Baigent et al., 2011).

Finally, glucose exposure was associated to technique failure. The increased risk fora definite transfer to hemodialysis for patients receiving higher intraperitoneal glucose load varied from 36 to 84% depending of the model and subgroup analyzed (Figure 2). The progressive increase observed for ultrafiltration failure as the cause of technique failure reinforce the negative impact of the glucose exposure on the peritoneal membrane permeability (Figure 3; Fusshoeller, 2008). However, our findings are in contrast to the study of Yang et al. in a cohort of 193 Canadian patients (Yang et al., 2008). This likely reflect differences in the management of volume status, for example the peritoneal equilibration test was not routinely performed in some centers due financial limitations. Of note, icodextrin was not available during the study period and could have helped to improve the management of hypervolemia.

Our study have some limitations including all those related to any cohort study. In addition, the lack of data on peritoneal membrane transport, longitudinal data on residual renal function and other unmeasured confounders including biomarkers of cardiovascular disease should also be taken into account in the interpretation of our results. Nevertheless, this is the largest cohort study to evaluate the association of glucose exposure with technique failure and patient survival. Moreover, both events preclude the occurrence of each other and no previous study of our knowledge considered the presence of competing risks in the investigation of the effect of peritoneal glucose exposure in these outcomes.

In conclusion, peritoneal glucose exposure was not associated with mortality but significantly affected technique failure.

## Author Contributions

All authors contributed to conception and design of the study and to manuscript revision, read and approved the submitted version. VR and TPM organized the database. TPM performed the statistical analysis and wrote the first draft of the manuscript.

## Conflict of Interest Statement

RP-F received research grants, consulting fees, and speaker honorarium from Baxter Healthcare. AF, PB, and TPM received consulting fees and speaker honorarium from Baxter Healthcare. The remaining author declares that the research was conducted in the absence of any commercial or financial relationships that could be construed as a potential conflict of interest.

## Supplementary Material

The Supplementary Material for this article can be found online at: https://www.frontiersin.org/articles/10.3389/fphys.2019.00150/full#supplementary-material
