# Supplementary material for: Impact of Glucose Exposure on Outcomes of a Nation-Wide Peritoneal Dialysis Cohort – Results of the BRAZPD II Cohort
Source: Front Physiol. 2019 Mar 5;10:150. doi: 10.3389/fphys.2019.00150 (PMC6411763; doi:10.3389/fphys.2019.00150)
Supplement: Supplementary file 1 [file Table_1.DOCX]

**Table S1.** Univariate Analysis of potential confounders for Technique Failure

|  | **Cox Regression** | | | **Competing Risk analysis** | | |
| --- | --- | --- | --- | --- | --- | --- |
| **Variables** | **HR** | **95% CI** | **P** | **HR** | **95% CI** | **P** |
| Residual Renal Function (yes) | 0.96 | 0.79 - 1.15 | 0.65 | 0.98 | 0.82 - 1.18 | 0.89 |
| Age >=65 | 0.78 | 0.64 - 0.95 | < 0.05 | 0.68 | 0.56 - 0.82 | < 0.05 |
| BMI < 18.5 | 1.02 | 0.93 - 1.11 | 0.62 | 1.01 | 0.93 - 1.11 | 0.43 |
| Previous hemodialysis (yes) | 1.22 | 1.03 - 1.46 | < 0.05 | 1.18 | 0.99 - 1.40 | < 0.05 |
| Peripheral Artery Disease (yes) | 0.95 | 0.76 - 1.19 | 0.70 | 0.86 | 0.68 - 1.07 | 0.19 |
| Diabetes (yes) | 1.04 | 0.87 - 1.24 | 0.59 | 0.95 | 0.80 - 1.13 | 0.62 |
| Coronary artery disease (yes) | 1.00 | 0.81 - 1.24 | 0.94 | 0.96 | 0.78 - 1.19 | 0.76 |
| Left Ventricular Hypertrophy (yes) | 1.07 | 0.89 - 1.29 | 0.44 | 1.08 | 0.90 - 1.30 | 0.35 |
| Heart Failure (yes) | 0.91 | 0.50 - 1.67 | 0.78 | 0.80 | 0.44 - 1.46 | 0.48 |
| Lupus (yes) | 1.39 | 0.80 - 2.41 | 0.23 | 1.39 | 0.81 - 2.38 | 0.21 |
| Stroke (yes) | 1.20 | 0.57 - 2.54 | 0.62 | 1.10 | 0.51 - 2.34 | 0.80 |
| Hypertension (yes) | 0.98 | 0.80 - 1.20 | 0.87 | 0.99 | 0.81 - 1.21 | 0.98 |
| HIV (yes) | 0.32 | 0.04 - 2.34 | 0.26 | 0.38 | 0.05 - 2.81 | 0.34 |
| Male gender | 1.00 | 0.84 - 1.19 | 0.97 | 1.00 | 0.84 - 1.19 | 0.93 |
| White Race | 0.86 | 0.72 - 1.03 | 0.19 | 0.83 | 0.69 - 0.99 | 0.92 |
| Family income (>2 MW) | 0.47 | 0.17 - 1.27 | 0.34 | 0.48 | 0.18 - 1.26 | 0.13 |
| Literacy (> 4 years) | 0.64 | 0.46 - 0.90 | 0.45 | 0.66 | 0.47 - 0.93 | 0.49 |

Legend: BMI = Body Mass Index; HIV = Human Immunodeficiency Virus; MW= Brazilian minimum wage

**Table S2.** Univariate Analysis of potential confounders for Patient Survival.

|  | **Cox Regression** | | | **Competing Risk analysis** | | |
| --- | --- | --- | --- | --- | --- | --- |
| **Variables** | **HR** | **95% CI** | **P** | **HR** | **95% CI** | **P** |
| Residual Renal Function (yes) | 0.79 | 0.68 - 0.92 | < 0.05 | 0.78 | 0.67 – 0.91 | < 0.05 |
| Age >=65 | 2.34 | 2.03 - 2.70 | < 0.05 | 2.44 | 2.12 – 2.82 | < 0.05 |
| BMI < 18.5 | 1.51 | 1.18 – 1.95 | <0.05 | 1.53 | 1.19 – 1.98 | < 0.05 |
| Previous hemodialysis (yes) | 1.18 | 1.02 - 1.36 | < 0.05 | 1.16 | 1.01 – 1.34 | < 0.05 |
| Peripheral Artery Disease (yes) | 1.87 | 1.60 – 2.18 | < 0.05 | 1.86 | 1.60 – 2.17 | < 0.05 |
| Diabetes (yes) | 1.83 | 1.59 – 2.12 | < 0.05 | 1.81 | 1.57 – 2.09 | < 0.05 |
| Coronary artery disease (yes) | 1.41 | 1.20 – 1.65 | 0.19 | 1.43 | 1.22 – 1.67 | 0.18 |
| Left Ventricular Hypertrophy (yes) | 1.12 | 0.96 – 1.30 | 0.12 | 1.14 | 0.98 – 1.33 | 0.07 |
| Heart Failure (yes) | 1.42 | 0.95 – 2.12 | 0.18 | 1.37 | 0.92 – 2.04 | 0.11 |
| Lupus (yes) | 0.98 | 0.58 – 1.67 | 0.95 | 0.95 | 0.57 – 1.34 | 0.86 |
| Stroke (yes) | 1.39 | 0.78 – 2.46 | 0.25 | 1.36 | 0.74 – 2.42 | 0.31 |
| Hypertension (yes) | 1.04 | 0.88 – 1.22 | 0.62 | 1.05 | 0.89 – 1.24 | 0.51 |
| HIV (yes) | 0.96 | 0.36 – 2.58 | 0.94 | 1.01 | 0.42 – 2.86 | 0.84 |
| Male gender | 1.00 | 0.86 – 1.15 | 0.96 | 1.00 | 0.86 – 1.15 | 0.92 |
| White Race | 1.23 | 1.06 – 1.44 | < 0.05 | 1.25 | 1.07 – 1.46 | < 0.05 |
| Family income (>2 MW) | 0.64 | 0.21 - 1.45 | 0.44 | 0.67 | 0.24 – 1.35 | 0.41 |
| Literacy (> 4 years) | 0.68 | 0.58 – 0.80 | < 0.05 | 0.67 | 0.58 – 0.79 | < 0.05 |

Legend: BMI = Body Mass Index; HIV = Human Immunodeficiency Virus; MW= Brazilian minimum wage
